# Microstructural Characterization of Cellulose Nanocrystals and Microcellulose from Bamboo (*Bambusa longispatha*) for Reinforcing Ordinary Portland Cement Matrix

**DOI:** 10.3390/polym16243558

**Published:** 2024-12-20

**Authors:** Parichat Thipchai, Kittisak Jantanasakulwong, Choncharoen Sawangrat, Jonghwan Suhr, Kittiphat Khotchapong, Pitiwat Wattanachai, Pornchai Rachtanapun

**Affiliations:** 1 Nanoscience and Nanotechnology, Faculty of Science, Chiang Mai University, Chiang Mai 50200, Thailand; parichat.t245@gmail.com; 2 Division of Packaging Technology, School of Agro-Industry, Faculty of Agro-Industry, Chiang Mai University, Chiang Mai 50100, Thailand; jantanasakulwong.k@gmail.com; 3 Center of Excellence in Agro Bio-Circular-Green Industry (Agro BCG), Chiang Mai University, Chiang Mai 50100, Thailand; 4 Department of Industrial Engineering, Faculty of Engineering, Chiang Mai University, Chiang Mai 50200, Thailand; choncharoen@step.cmu.ac.th; 5 School of Mechanical Engineering, Sungkyunkwan University 2066 Seobu-ro, Jangan-gu, Suwon-si 16419, Republic of Korea; suhr@skku.edu; 6 Department of Civil Engineering, Faculty of Engineering, Chiang Mai University, Chiang Mai 50200, Thailand; kittiphat_koch@cmu.ac.th

**Keywords:** cement composites, natural fibers, nanocellulose, compressive strength, flexural strength

## Abstract

This study investigates the microstructural characterization of cellulose nanocrystals (CNC) and microcellulose (MC) extracted from bamboo fibers (*Bambusa longispatha*) and their potential as reinforcement agents in ordinary Portland cement (OPC) composites. CNC with a mean particle size of 29.3 nm and MC with a mean size of 14.6 × 10^3^ nm were incorporated into OPC at varying concentrations (0.1%, 0.2%, 0.4%, and 0.6% by cement mass). The compressive strength analysis revealed that increasing MC content led to a decrease in strength, with reductions ranging from 8.8% to 25.9% relative to the control OPC, while the CNC-enhanced composite at 0.4% achieved the highest compressive strength of 43.2 MPa. Flexural strength analysis indicated a minor increase in strength with MC addition (from 7.5 MPa to 8.1 MPa), while CNC addition at 0.1% improved flexural strength to 8.2 MPa but declined with higher concentrations. SEM and stereo microscopy demonstrated MC and CNC dispersion and highlighted microstructural differences, including pore distribution in the composites. XRD analysis showed increased crystallinity for CNC composites compared to pure OPC, with the highest crystallinity index of 52.2% observed at 0.4% CNC. This study highlights that CNC at specific concentrations can enhance OPC mechanical properties, while higher MC and CNC additions may impact strength properties variably due to their microstructural integration and crystallinity. These findings support the potential for bamboo-derived cellulose materials in enhancing cementitious composite performance.

## 1. Introduction

The increasing demand for sustainable materials in construction, driven by the need to reduce the environmental impact of traditional building materials, has spurred interest in utilizing natural fibers as reinforcements in cementitious composites [1]. Natural fiber-reinforced cement composites improve the performance of structural components by reducing the brittleness of regular cement and enhancing its impact strength and flexibility [2]. Furthermore, since natural fibers are abundant and of high quality, they offer an affordable and environmentally responsible alternative for reinforcing cement in agriculture [3]. Natural fibers such as sugarcane bagasse [4], flax [5], hemp [2], jute [6], sisal [7], and bamboo [8] are becoming popular as sustainable options for making cement materials. These natural fibers are renewable and help reduce the carbon footprint of building materials, supporting global sustainability goals [9]. Among these natural fibers, bamboo (*Bambusa longispatha*) has garnered particular attention as a promising source of cellulose for reinforcing cement matrices [10]. Bamboo grows quickly and is strong and adaptable, making it a great source of cellulose. It is commonly used in furniture, handicrafts, and construction [11,12,13].

Cellulose, the main structural component of plant cell walls, is a linear chain of glucose units connected by β-(1-4)-D-glucopyranose, extracted from natural fibers for diverse applications [14]. Cellulose was extracted through pulping with an alkaline solution, followed by bleaching and optional grinding into microcellulose (MC). MC is lightweight, strong, biodegradable, and flexible, with high water absorption, and moderate thermal stability, making it ideal for composites and industrial applications [15]. When MC is hydrolyzed with acid, it produces cellulose nanocrystals (CNC), which have unique properties such as small size, high crystallinity, large surface area, and excellent mechanical strength [10]. These cellulose derivatives hold significant potential for improving the mechanical properties of cement composites, enhancing their strength and durability, and reducing brittleness [16,17]. The extraction of cellulose derivatives, including MC and CNCs, has become a focal point in exploring the versatility of bamboo fibers. The application of these cellulose derivatives in OPC cement matrices has been shown to improve mechanical performance and durability [18].

Numerous studies have explored the integration of MC and CNC reinforcements into OPC matrices [18,19,20]. MC-reinforced cement composites improve toughness, ductility, flexural strength, and fracture resistance. The fibers bridge cracks, enhancing load transfer and post-cracking performance, making the composite stronger and more durable for construction use [21]. In OPC composites, Hamada et al. [22] found that natural MC improved the bonding with the cement matrix, enhancing the toughness of MC-reinforced cement composites. Similarly, Gao et al. [23] found that adding sisal fiber to concrete improved the fracture behavior of cement-based materials, increasing the flexural strength by 16.7%. According to Nassiri et al., CNC, being short and rod-shaped, exhibits high mobility, which effectively reduces viscosity, enhances stability, and functions as a curing retardant for cement [24]. Guo et al. found that CNC enhances cement density, reduces water absorption, delays hydration initiation, improves mechanical properties, and refines pore structure [25]. In addition, Arif Aziz et al. [26] reported that the incorporation of CNC as additives in mortar enhanced compressive strength and reduced void volume. The application of these cellulose derivatives in OPC cement matrices has been shown to improve mechanical performance and durability.

Bamboo fiber-reinforced composites show promise, but research on using nano- and micro-scale cellulose as reinforcement in OPC remains limited [27]. Despite advancements in bamboo (*Bambusa longispatha*) cellulose extraction and characterization, gaps persist in understanding how specific concentrations and particle sizes of MC and CNC affect the mechanical properties, particularly the compressive and flexural strength variations of OPC cement. Therefore, this study focuses on extracting MC and CNC from bamboo fibers and incorporating them as reinforcement materials in OPC cement composites. MC and CNC were added at varying concentrations of 0.1%, 0.2%, 0.4%, and 0.6% by cement mass to evaluate their effects on the chemical and mechanical properties of the composites. In addition to examining compressive and flexural strength, this research investigates the impacts of cellulose microstructure and crystallinity on cement hydration and phase distribution within the composite. The findings provide valuable insights into the effective use of bamboo-derived cellulose to enhance the performance of OPC cement matrices.

## 2. Materials and Methods

### 2.1. Materials

General purpose TPI OPC type I, compliant with ASTM C150 and TIS 15-1, with a specific gravity of 3.13, was used. Local river sand was served as a fine aggregate (unit weight: 1509 kg/m^3^, fineness modulus: 2.90, gradation: ASTM C778). Bamboo culms (*Bambusa longispatha*) were obtained from Samoeng District, Chiang Mai Province, Thailand. AR-grade chemicals, including sodium hydroxide, glacial acetic acid, sodium chlorite, and sulfuric acid, were sourced from Merck (Darmstadt, Germany) for MC and cellulose CNC extraction.

### 2.2. Extraction of MC and CNC

**Figure 1:**
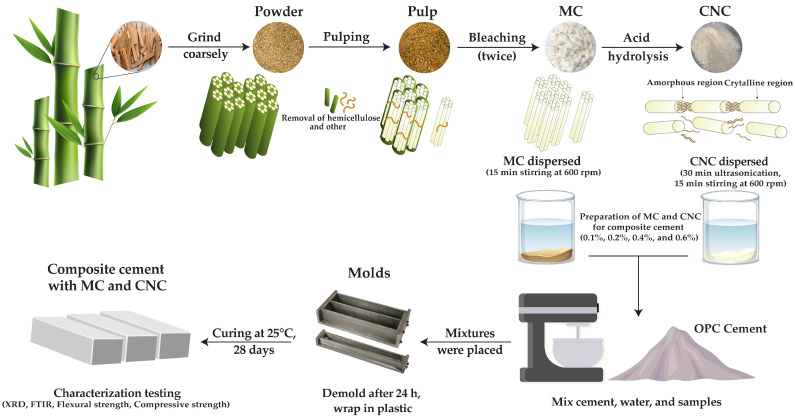
Experimental design of MC and CNC extraction from bamboo fiber and preparation of cement composites with MC and CNC.

In Figure 1, MC and CNC were extracted from raw bamboo fiber by removal of hemicellulose and other components through alkaline treatment and sodium chlorite bleaching. Subsequently, the obtained MC was hydrolyzed using sulfuric acid, following the method described by Thipchai et al. [28]. In brief, 500 g of bamboo powder was boiled in a solution with 18% *w*/*v* sodium hydroxide for 5 h at 85 °C and continuously stirred (Overhead Stirrers, Bethai Bangkok Equipment & Chemical, Bangkok, Thailand). The dried pulp was then bleached twice using a 3.4% *w*/*v* sodium chlorite solution prepared with an acetate buffer (acetic acid and sodium chlorite) at 85 °C for 3 h. This process was repeated twice to obtain MC. The final products were sieved using an 80-mesh sieve. The obtained MC was then further processed to extract CNC. The obtained MC was then further extracted to produce CNC. MC (50 g) was hydrolyzed using 32% (*v*/*v*) sulfuric acid at 50 °C for 5 h with continuous stirring. The sample was initially adjusted to pH 7 using distilled water, followed by the addition of a 0.5% *w*/*v* sodium hydroxide solution. The solution was blended using a high-speed blender (SBD12A, Anitech, Nonthaburi, Thailand) at 36,000 rpm for 30 min. The CNC suspension was sonicated in an ice bath for 30 min at 20 kHz using an ultrasonic generator (BEM-900A, Bueno-Biotech, Nanjing, China). The sample was then stored at −20 °C for 24 h and freeze-dried at −60 °C for 48 h using a freeze-dryer (DW-10N, Drawell Instrument, Chongqing, China). Finally, dried MC and CNC were characterized.

### 2.3. Preparation of Cement Composites with MC and CNC

Begin by preparing the water required for mixing the cement, maintaining a water-to-cement (w/c) ratio of 0.3. MC and CNC powders were added to the water to ensure proper dispersion. MC powder was dispersed in the water to prevent clumping, using continuous mechanical stirring at 600 rpm for 15 min. Meanwhile, CNC powder was dispersed in the water to create a stable suspension through ultrasonication for 30 min, followed by continuous mechanical stirring at 600 rpm for another 15 min. Cement mixtures were prepared by incorporating MC and CNC at rates of 0.1%, 0.2%, 0.4%, and 0.6% by mass of cement. The water containing dispersed MC and CNC was gradually added into the OPC cement using a mechanical mixer (Hobart A200, Hobart, London, UK) to ensure thorough and homogeneous mixing of the cement, water, MC, and CNC. Following thorough mixing, the prepared cement mixtures were poured into 4 cm × 4 cm × 16 cm prism molds. After 24 h of curing in the molds, the specimens were carefully demolded, wrapped in plastic film, and subsequently cured at room temperature until they reached the designated testing age. The details of the mixture compositions and their respective proportions are provided in Table 1.

### 2.4. Compressive Strength Analysis

After 28 days of room-temperature curing, the compressive strength of the 5 cm × 5 cm × 5 cm cement composite specimens was measured. Five specimens from each batch were tested using a compression testing machine (YES-300 Digital Display Compression Testing Machine, Jinan Hensgrand Instrument Co., Ltd., Jinan, China) at a constant loading rate of 300 kN. The average compressive strength values were calculated and reported in accordance with ASTM C109 [29].

### 2.5. Flexural Strength and Flexural Toughness Analysis

The 4 cm × 4 cm × 16 cm specimens of cement composites were prepared following the BS-EN 196-1 standard [2]. Flexural strength tests were conducted using a 250 kN control universal testing machine (UTM) (Digital Tritest 50 Load Frame, ELE International, Loveland, CO, USA) at 28 days. All measurements were performed in triplicate. The flexural strength of cement composites was calculated using Formula (1):

(1) $$\sigma f=\frac{3P_{max}L}{2bd^{2}}\;$$

where *σf* is the flexural strength (MPa), *P_max_* is the maximum load (N), *L* is the length of the beam between supports (mm), *b* is the width of the beam (mm), and *d* is the depth (thickness) of the beam (mm).

Flexural toughness parameters were calculated as the area under the load–deflection curve, according to ASTM C1609 method [30].

### 2.6. Morphological Analysis

The morphology and microstructure of cement composites were analyzed using a Leica S8 APO Greenough stereo microscope (Leica Microsystems, Wetzlar, Germany), scanning electron microscopy (SEM) equipped with an energy-dispersive X-ray spectrometer (EDS) (JSM-IT200, JEOL, Tokyo, Japan) for MC, and field emission scanning electron microscope (FE-SEM) with STEM capability (FE-SEM, JSM-IT800, JEOL, Tokyo, Japan) for CNC. To prepare the samples, small pieces machined from the flexural test samples were immersed in acetone for 24 h. Dried samples were mounted on a brass stub with a carbon tab and coated with a gold layer via sputtering for 30 s. The SEM analysis was conducted at a voltage of 15 kV.

**Figure 2:**
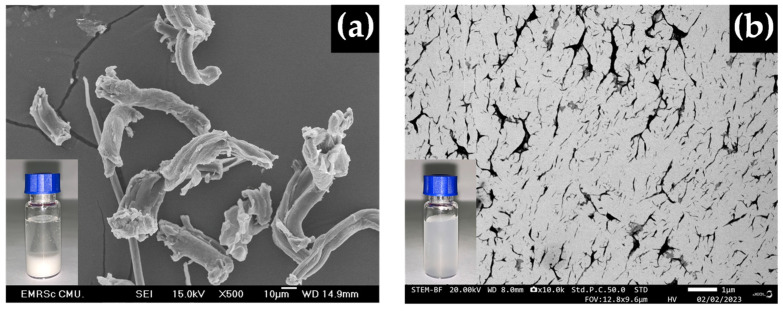
SEM of bamboo fibers from (**a**) MC at 500× and (**b**) CNC at 10,000×.

Figure 2 shows SEM images of MC and CNC. MC exhibits a large fibrous morphology with average dimensions in the micron range, while CNC displays a nanocrystalline morphology with an average diameter up to 1000 times smaller than that of MC, as detailed in Table 2.

### 2.7. Fourier Transform Infrared (FTIR) Analysis

The FTIR spectrometer (FTIR-4700, JASCO International, Pfungstadt, Germany) was used to evaluate the functional groups of MC, CNC, and cement composites. The powdered samples, weighing approximately 2 mg, were crushed, mixed with KBr, and then compressed into pellets for examination. FTIR measurements in transmission mode were carried out between 500 cm^−1^ and 4000 cm^−1^.

### 2.8. X-Ray Diffraction (XRD) Analysis

The mineral compositions of cement composites were characterized using XRD analysis (X-ray Diffractometer, Miniflex 600, Rigaku, Selangor, Malaysia). Samples were scanned at a rate of 2° per min with a scattering angle (2θ) ranging from 5° to 60°. To stop hydration, the samples were immersed in acetone for 24 h. Afterward, they were dried and ground into powders that passed through an 80-mesh sieve. Additionally, the crystallographic structure and crystallinity of the MC and CNC samples were evaluated using XRD patterns. The crystallinity index and crystallite size were calculated based on our previous report [31].

### 2.9. Statistical Analysis

Statistical analysis was performed using SPSS software (version 16.0, SPSS Inc., Chicago, IL, USA), and all data were presented as means with standard deviations. One-way ANOVA was utilized to assess a significance criterion of *p* < 0.05.

## 3. Results and Discussion

### 3.1. Effect of MC and CNC on Compressive Strength

**Figure 3:**
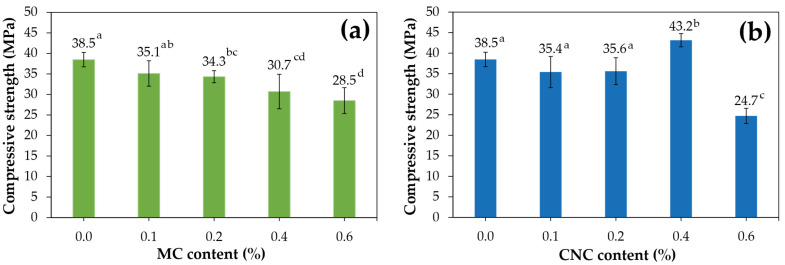
Compressive strength of cement composites at 28 days for (**a**) MC and (**b**) CNC with varying contents. The results are shown as mean ± standard deviation; a, b, and c (*p* < 0.05).

In comparison to OPC samples made without MC and CNC, Figure 3 presents the compressive strength results of cured mortar samples reinforced with two distinct types of bamboo fibers at 28 days, with varying contents of 0.1%, 0.2%, 0.4%, and 0.6%. Figure 3a demonstrates that the compressive strength decreases as the MC contents increase from 0.1–0.6%, resulting in a reduction in compressive strength of 8.8–25.9% compared to the OPC samples without MC. At higher contents, cellulose fibers tend to agglomerate, forming clusters that create weak points, reducing strength. Additionally, they increase porosity, leading to more voids in the matrix, which weakens the composite cement’s ability to bear compressive loads [32]. This finding is consistent with studies on cement mixes including hemp fiber, which found that adding 2% to 4% hemp fiber decreased the compressive strength of the cement in comparison to cement without fiber [2]. Figure 3b indicates a slight decrease in compressive strength for cement composites with CNC content of 0.1–0.2%, resulting in reductions of 7.4–8.0% compared to OPC. This decrease is due to the ineffective dispersion of CNC at lower concentrations, resulting in a less uniform matrix and weaker reinforcement, which weakens the material [24]. In contrast, the 0.4% CNC mixture exhibited the highest compressive strength at 43.2 MPa, reflecting a 12.2% reduction compared to the same OPC samples. The compressive strength decreases as the CNC contents increase to 0.6% at 24.7 MPa. The addition of 0.4% CNC provided a denser matrix with lower porosity, resulting in the highest increase in compressive strength [18]. In addition, the optimum concentration of 0.4% CNC in cement composites enhances compressive strength by promoting effective dispersion and interaction with the cement matrix, which facilitates moisture absorption within the cement phase. This concentration balances between reinforcement and dispersion, preventing agglomeration that could otherwise weaken the material. The hydrophilic property of CNC’s nature accelerates cement hydration, leading to the formation of larger volumes of hydration products. Its high specific surface area strengthens the CNC–matrix interface, improving stress transfer between the matrix and CNC. The high surface area to volume ratio of CNC promotes the nucleation of cement hydration phases such as calcium silicate hydrate (CSH), which further accelerates hydration. However, increasing CNC concentration to 0.6% causes agglomeration, poor dispersion, increased viscosity, and reduced strength due to CNC’s intrinsic physical properties, particularly the high number of hydroxyl groups, which create strong hydrogen bonds between CNC, leading to aggregation [33]. These experimental results are consistent with the findings of Aziz et al. [26], which indicate that the optimal compressive strength of CNC-based mortar is achieved with CNC content between 0.25% and 0.50%. Similar to our previous research, this study found that at 0.4% CNC, cassava starch composite films exhibited increased strength, but at 0.6%, agglomeration caused a decrease in compressive strength [28]. Previous research on the relationship between relative compressive strength and fiber addition percentages found that excessive fibers can disrupt the interactions between cement particles. Additionally, the agglomeration of nanomaterials leads to a reduction in mechanical strength [18,34]. CNC improves uniformity by filling nanoscale pores and preventing the formation of cracks that can lead to the growth of micro-cracks. Moreover, its high water absorption capacity enhances adhesion to cement and promotes strength properties [35]. To improve MC and CNC dispersion in cement composites, methods like TEMPO-mediated oxidation, ultrasonication, high-shear mixing, and pre-dispersion in solvents can be employed. These techniques reduce agglomeration and enhance performance, but they may increase production costs and processing time, requiring a balance between cost and efficiency [33].

### 3.2. Effect of MC and CNC on Flexural Strength

**Figure 4:**
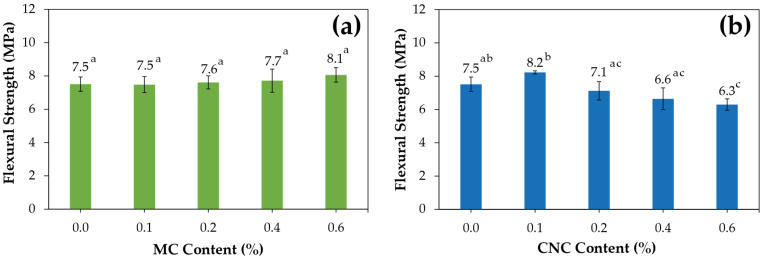
Flexural strength of cement composites at 28 days for (**a**) MC and (**b**) CNC with varying contents. The results are shown as mean ± standard deviation; a, b, and c (*p* < 0.05).

**Figure 5:**
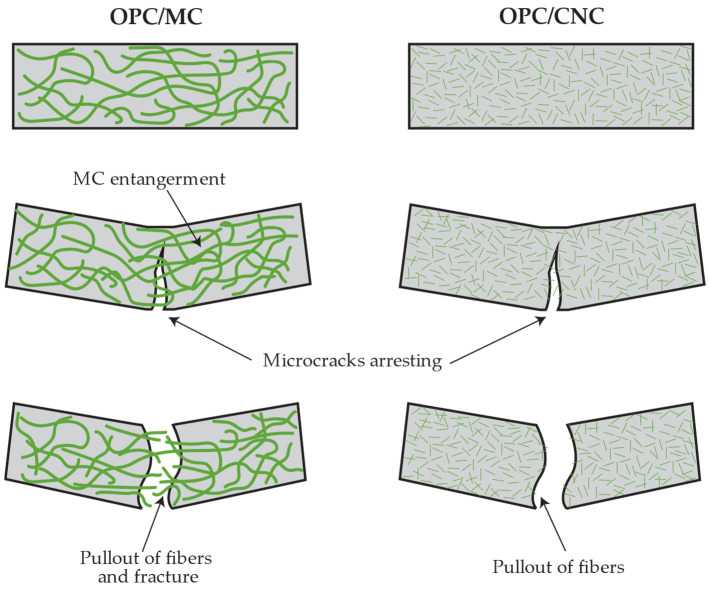
Illustrations of fracture mechanisms of OPC/MC and OPC/CNC cement composites.

Flexural testing was conducted on various cement mortar samples reinforced with bamboo fibers (MC and CNC) at 28 days, along with control samples without fibers. The experimental results indicated that with the addition of MC in amounts ranging from 0.1% to 0.6%, as shown in Figure 4a, the flexural strength increased slightly from 7.5 MPa to 8.1 MPa. In contrast, the addition of CNC led to an increase in flexural strength to 8.2 MPa at a 0.1% addition, followed by a gradual decrease to 7.1 MPa at 0.2%, 6.6 MPa at 0.4%, and 6.3 MPa at 0.6%, as shown in Figure 4b. This result aligns with findings by Onuaguluchi et al., showing CNC addition enhances flexural strength and energy absorption in cement, with peak performance at 0.1% CNC [36]. In addition, the results of this research tend to be consistent with a study on the effect of cellulose from softwood fiber content (5% to 10%) on cement composites, which found that flexural strength ranged from 10 to 20 MPa and slightly increased with higher fiber content. The effect of cellulose characteristics (softwood and hardwood) revealed that softwood fibers, with their longer lengths, provided a more balanced improvement in flexural strength compared to shorter hardwood fibers [20]. Similarly, MC has a longer fiber length than CNC, as shown in Table 2. Adding an appropriate amount of MC enables the formation of an entanglement network that enhances reinforcement in the cement matrix and increases flexural strength by distributing force. However, excessive addition can lead to micro-crack formation, negatively impacting overall strength [2,37]. The failure mechanisms of OPC when reinforced by CNC and MC are depicted in Figure 5. MC fibers induce a microcrack-arresting mechanism. Their micrometric dimensions contribute to the arrest of microcracks, preventing their propagation. The lengths and network structures of the fibers promote visible pullout along fractured surfaces, enhancing crack-arresting behavior within the cement composite. In addition, the research by Sambucci et al. [38] reported an important improvement mechanism induced by cellulose, specifically the increased flexural strength of cement with MC. This enhancement is attributed to its hygroscopic properties, which facilitate moisture absorption and improve interfacial adhesion and stress transfer in cement composites. The observed increase in compressive strength is attributed to strong hydrogen bonding between OPC and CNC, as well as the relatively uniform dispersion of fibers facilitated by robust interfacial interactions. Enhanced strength and strain-at-failure contribute to the fracture toughness of P-CNC0.4 composites, which exceeds that of the OPC control [3]. The results of cement composites from CNC align with previous studies, where CNC additions of 0.1–0.2% improved flexural strength by effectively mitigating the porosity of OPC cement, significantly enhancing its mechanical performance and durability [39]. However, at higher CNC contents, flexural strength declined. This reduction was due to the high crystallinity of CNC, which increased brittleness, and the tendency of nano-sized CNC particles to agglomerate at higher contents, reducing flexural strength [18].

**Figure 6:**
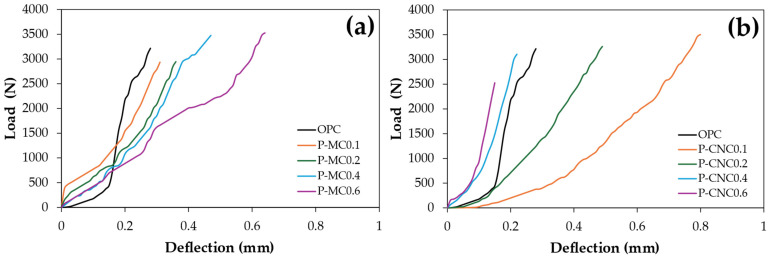
Load versus deflection of cement composites at 28 days for (**a**) MC and (**b**) CNC with varying contents.

Figure 6 presents the load versus deflection curves for cement composite specimens under flexural testing, highlighting their distinct structural responses. The OPC control represents brittle failure, where the material fractures or fails once the load reaches a critical point [40]. The samples, reinforced with 0.1%, 0.2%, 0.4%, and 0.6% bamboo fibers in both MC and CNC, were compared to the control mixtures. The analysis of load versus deflection revealed that the inclusion of higher percentages of MC significantly increased the load versus deflection. At the 0.6% reinforcement (3526.0 N at 0.5 mm), MC provided superior load resistance compared to OPC and CNC, as shown in Table 3. However, reinforcement with 0.1% CNC demonstrated the highest deflection, reaching 3504.3 N at 0.8 mm, with a gradual decrease in deflection as the CNC content increased to 0.2%, 0.4%, and 0.6%, respectively. Similarly, the flexural toughness parameters of the cement composites increased progressively with the addition of MC at 0.1%, 0.2%, and 0.4%, reaching the highest flexural toughness of 992.9 N·mm at 0.6%. In contrast, CNC reinforcement initially enhanced flexural toughness at 0.1% (900.4 N·mm), but a decline was observed with higher content, as shown in Table 3. CNC enhances the toughness of composites compared to MC at 0.1% due to its nanoscale properties. The high surface area of CNC allows for stronger bonding with the cement matrix, improving stress transfer and energy absorption [33]. Additionally, CNC forms a dense, interconnected network within the matrix, which effectively distributes stress and arrests microcracks by bridging them and preventing their propagation. This nanoscale crack-arresting ability provides numerous closely spaced points to resist fracture. In contrast, long MC acts more as structural reinforcements that prevent brittle fracture but may not have the same ability to absorb and dissipate energy. They are effective at distributing macro-scale stress but may not interact at the nanoscale level as effectively as CNC, leading to lower toughness. The larger size and lower surface area of the MC reduce their efficiency in enhancing the energy absorption capacity compared to CNC [41]. Compared to the OPC control, the flexural toughness of CNC-reinforced composites at 0.4% and 0.6% was lower than that of the OPC control. Previous findings on the influence of fiber type indicate that natural fibers contribute to a balanced enhancement in toughness [20]. Cement composites containing randomly distributed fibers (MC and CNC) exhibit enhanced performance compared to plain concrete, which fails suddenly after reaching its ultimate flexural strength. These fiber-reinforced composites can bear significant loads even after large deflections, preventing immediate failure after the first crack. This feature reduces fracture propagation, leading to an increase in toughness [30].

### 3.3. Morphological Analysis of Cement Composites 

**Figure 7:**
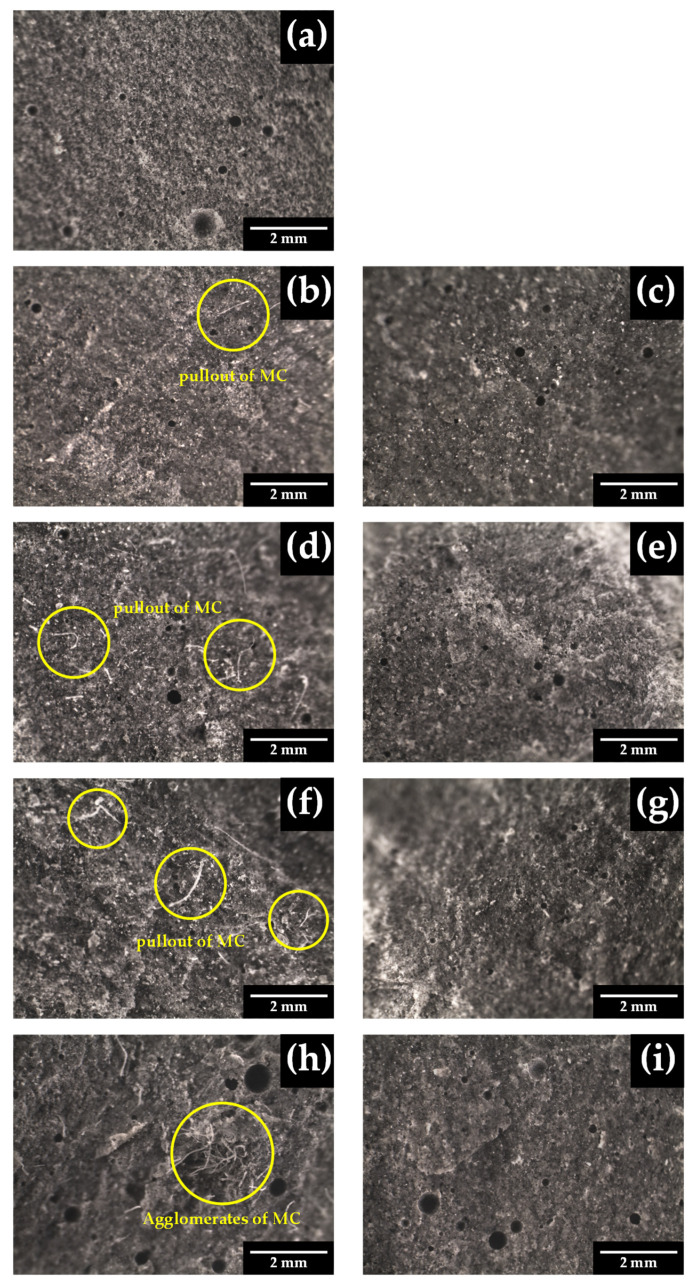
Morphology and physical appearance of cement composites at 28 days by stereo microscope at 16× magnification: (**a**) OPC, (**b**) P-MC0.1, (**c**) P-CNC0.1, (**d**) P-MC0.2, (**e**) P-CNC0.2, (**f**) P-MC0.4, (**g**) P-CNC0.4, (**h**) P-MC0.6, and (**i**) P-CNC0.6.

Figure 7 illustrates the morphology and physical appearance of cement composites after 28 days. Significant differences were observed among samples with varying MC and CNC contents of 0.1%, 0.2%, 0.4%, and 0.6%. Increasing the MC content from 0.1% to 0.6% led to a corresponding increase in porosity, aligning with the previously noted reduction in compressive strength (Figure 3). Furthermore, in the P-MC0.6 composite, cellulose formed an entanglement network, enhancing both flexural strength and toughness (Figure 4 and Table 3). This effect is due to MC absorbing water, creating micro-voids between the fibers and the matrix, and tending to form agglomerates [42]. Furthermore, MC can entrap air within the mix, resulting in higher porosity and reduced compressive strength. Nevertheless, cellulose can improve flexural toughness by microcracks arresting in the composite [3,20]. Cement composites reinforced with CNC at concentrations of 0.1–0.2% exhibited surface porosity similar to that of the control OPC. However, P-CNC0.4 (Figure 7g) reduced porosity due to the small molecular size of CNC. Proper distribution of CNC enhances cement density by filling micro-pores, improving particle packing, and strengthening the bond with the cement matrix [43]. As a result, the structure becomes more cohesive with fewer voids. Furthermore, CNC assists in regulating water retention, reducing the formation of large pores during curing [26]. On the other hand, at 0.6%, the aggregation of CNC led to a decrease in compressive strength, as shown in Figure 3.

**Figure 8:**
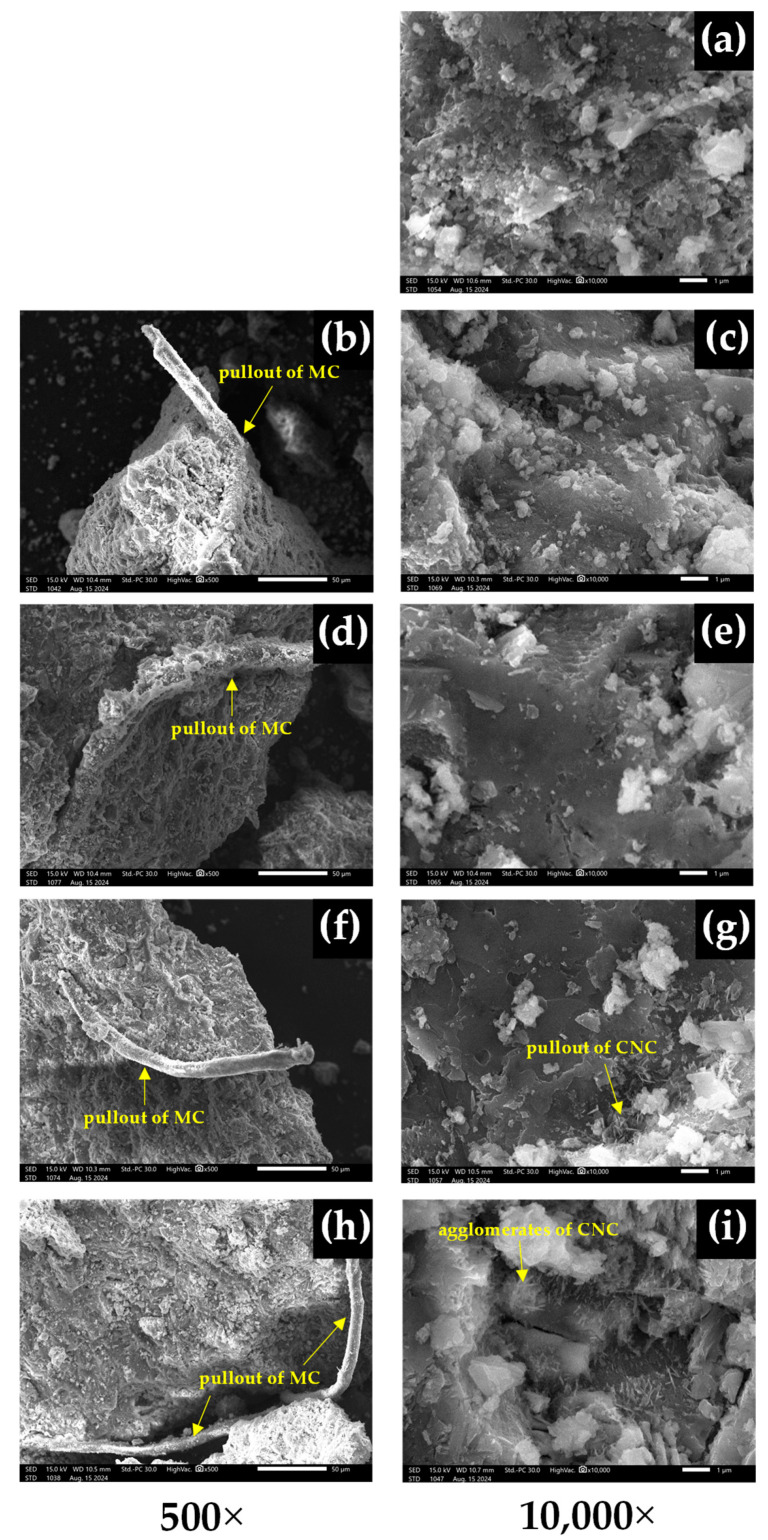
SEM micrographs of cement composites at 28 days, shown at 500× and 10,000× magnifications: (**a**) OPC, (**b**) P-MC0.1, (**c**) P-CNC0.1, (**d**) P-MC0.2, (**e**) P-CNC0.2, (**f**) P-MC0.4, (**g**) P-CNC0.4, (**h**) P-MC0.6, and (**i**) P-CNC0.6.

The SEM micrographs of the MC- and CNC-reinforced cement composites at concentrations of 0.1%, 0.2%, 0.4%, and 0.6% are shown in Figure 8. SEM observations in Figure 8b,d,f,h reveal the characteristics of MC in cement composites. At low contents, flexural stress failure is attributed to fiber fracture, as the fiber–matrix bond is strong enough to break the fibers. At high contents, an entanglement network of adjacent fibers forms, which reduces adhesion between the fibers and the matrix, leading to pore formation and ultimately causing fractures, as can be seen from Figure 5 and Figure 7h [2]. In this situation, the aspect ratio of the fibers plays a critical role in enhancing the toughness of the cement composite [20].

On the other hand, when comparing the cement composites reinforced with CNC to the control OPC, it was observed that the addition of CNC at concentrations of 0.1%, 0.2%, and 0.4% resulted in progressively smoother cement surfaces. Notably, the addition of CNC at 0.4% produced a more homogeneous dispersion and uniform microstructure. These findings align with the results of Santos et al. [18], who reported that nanocellulose concentrations between 0.2% and 0.4% in reinforced cement were more effective in reducing microcracks caused by expansion and water evaporation. Nevertheless, agglomeration of CNC-reinforced cement composites occurs at high contents of 0.6% due to their high surface area and strong intermolecular interactions, such as hydrogen bonding [28,35]. As content increases, dispersibility decreases, leading to clustering. Moreover, the increased hydrophilic nature of CNC contributes to the formation of agglomerates, which can be observed in Figure 8i [36]. This leads to higher compressive strength at 0.4% CNC and lower strength at 0.6% CNC.

**Figure 9:**
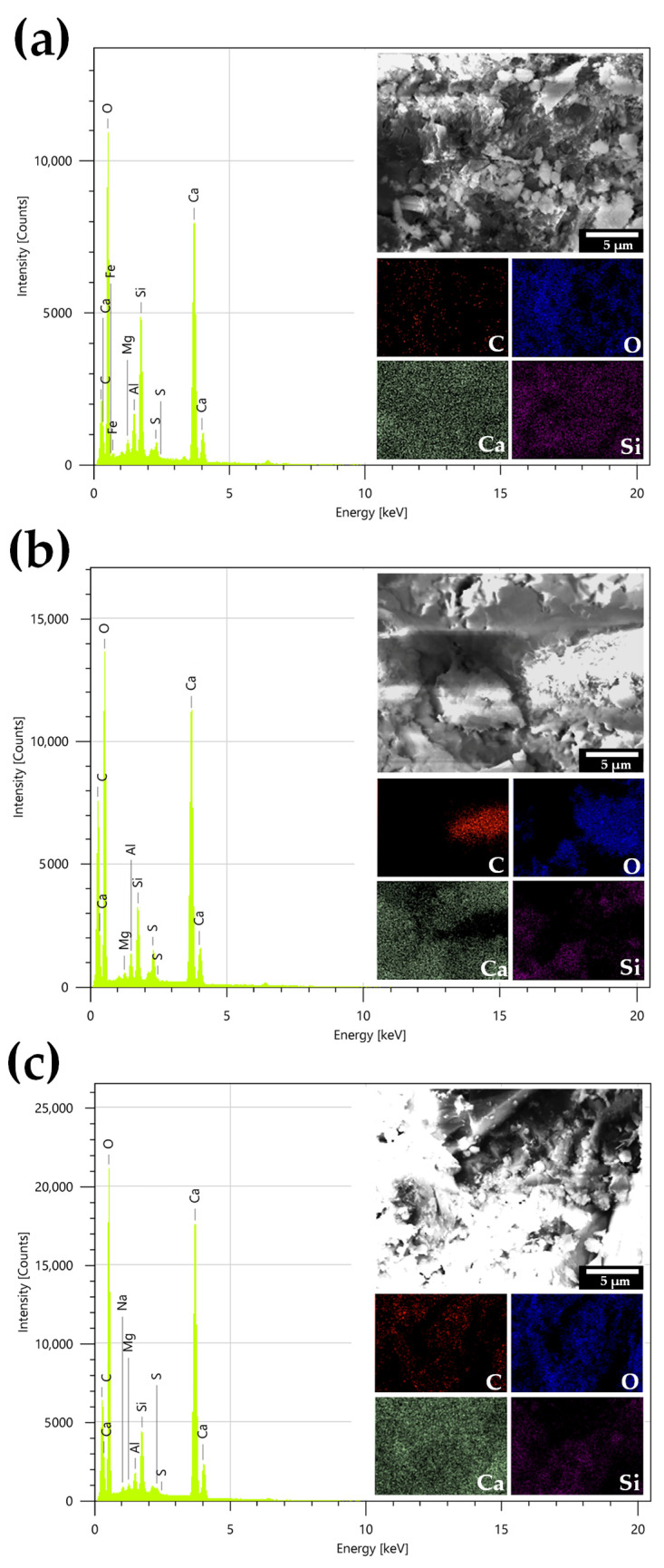
SEM diagrams of cement composites after 28 days, along with EDS spectra and element distribution maps for C, O, Ca, and Si: (**a**) OPC, (**b**) P-MC0.4, and (**c**) P-CNC0.4.

Figure 9 presents the SEM image and EDS spectra distribution maps of MC- and CNC-reinforced OPC cement composites. The primary mineral components of OPC, as shown in Figure 9a, include carbon (C), oxygen (O), calcium (Ca), silicon (Si), aluminum (Al), sulfur (S), and magnesium (Mg) [36]. Upon the addition of CNC to OPC (Figure 9c), the EDS spectra revealed a noticeable increase in C content, which is attributed to the presence of CNC. The distribution of C and O elements was relatively uniform across the composite, indicating that the CNCs were well dispersed throughout the cement matrix. In contrast, the distribution of MC elements, particularly C, appeared concentrated predominantly on the right side of the EDS map (Figure 9b). This suggests an uneven distribution of MC due to the larger particles. The overall test results confirmed that the addition of CNC did not significantly alter the distribution of major elements such as Ca, Si, or O within the cement composite. Furthermore, CNC was found to be uniformly distributed within the cement composite, which positively impacted the arrangement of the particles. This uniform dispersion of CNC contributes to enhanced packing of the particles and may reduce the overall porosity of the cement matrix. These observations suggest that while CNC enhances the structural arrangement, it does not alter the fundamental mineral composition of OPC. However, CNC can improve the overall microstructural properties, leading to potential benefits in improved mechanical properties and durability [26,39].

### 3.4. Functional Groups

**Figure 10:**
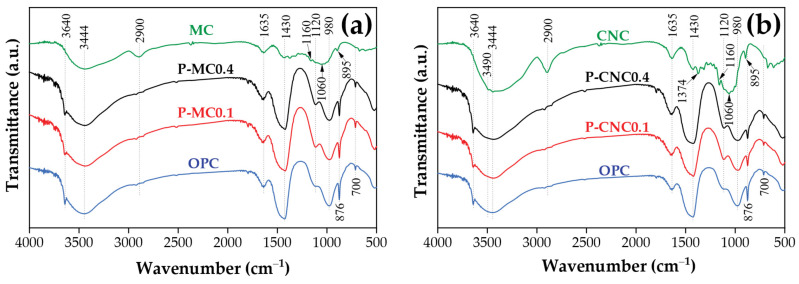
FTIR spectra of cement composites after 28 days: (**a**) MC and (**b**) CNC.

Figure 10 illustrates the FTIR analysis of MC, showing chemical changes following sulfuric acid hydrolysis to form CNC. Key absorption peaks include 3444 cm^−1^ (O–H stretching), 2900 cm^−1^ (C–H stretching), and 1635 cm^−1^ (H–O–H stretching of water) [44,45]. Figure 10b shows CNC peaks at 1160 cm^−1^, 1060 cm^−1^, and 895 cm^−1^, indicating the preserved cellulose structure [15]. Hydrolysis increased peak intensity in cellulose structure, while CNC showed narrow peaks at 1060 cm^−1^, 3444 cm^−1^, and 3490 cm^−1^ (O–H stretching in cellulose I and II) [31]. The FTIR spectra of cement composites reveal key bands with a sharp peak at 3640 cm^−1^ for calcium hydroxide (Ca(OH)_2_) and a broad peak near 3400 cm^−1^ and 2900 cm^−1^ for O–H stretching of hydroxyl groups water. The peak around 1635 cm^−1^ indicates bending vibrations of water (H–O–H), while peaks at 876 cm^−1^ and 700 cm^−1^ correspond to Si–O bending vibrations. Additionally, peaks near 1430 cm^−1^ are due to the C–O stretching of calcium carbonate (CaCO_3_), and the peak at 980 cm^−1^ indicates Si–O stretching related to calcium silicate hydrate (C–S–H). A band for S–O stretching appears near 1120 cm^−1^ and is partially obscured by the Si–O stretch [46]. However, the FTIR spectra of the cement composites containing MC and CNC at contents of 0.1% and 0.4% exhibited only slight differences compared to the spectra of OPC. This is primarily due to the strong signals from the OPC matrix, which can overshadow any subtle changes induced by the MC and CNC [47]. In addition, changes in peak intensity, particularly in the hydroxyl stretching region (3200–3600 cm^−1^), indicate interactions between CNC and OPC [3]. These shifts suggest hydrogen bonding or physical interactions between the hydroxyl groups in CNC and the OPC matrix, along with a relatively homogeneous fiber dispersion due to this strong interaction, which affects the strength of CNC-reinforced cement composites, as shown in Figure 3b [8].

### 3.5. Mineralogical and Crystallinity Analysis

**Figure 11:**
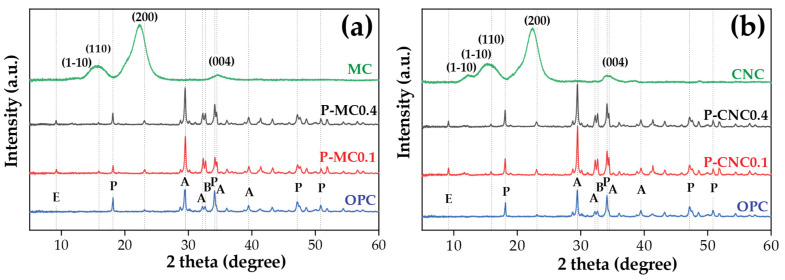
XRD spectra of cement composites after 28 days: (**a**) MC and (**b**) CNC. (A: tricalcium silicate (C_3_S), B: dicalcium silicate (C_2_S), E: ettringite, and P: portlandite).

The XRD patterns for MC and CNC are shown in Figure 11. CNC retained the cellulose I crystal structure, while cellulose exhibited peaks at 2θ = 15.4°, 16.4°, 22.5°, and 34.5°, corresponding to the (1–10), (110), (200), and (004) crystalline patterns. In contrast, CNC showed peaks at 2θ = 12.5°, 20.2°, 22.3°, and 34.5°, indicative of cellulose allomorph II structures. This shift from type I to type II in CNCs resulted from hydrolysis with sulfuric acid and subsequent mechanochemical and ultrasonic processing [28]. The XRD analysis of OPC control and the cement mortar samples reinforced with 0.1% and 0.4% bamboo fibers (MC and CNC) specimens after 28 days in sealed curing conditions. The XRD analysis of cement mortar samples reinforced with 0.1% and 0.4% bamboo fibers (MC and CNC) and OPC control samples following 28 days of sealed curing is shown in Figure 11. The main XRD peaks found are ettringite (E) at 2θ of 9.2° and portlandite (P) with 2θ values of 18.0°, 34.1°, 47.1°, and 50.8° [47]. Peaks in the 2θ range of 29.5°, 32.0°, 34.3°, and 39.5° are attributed to tricalcium silicate (A), while dicalcium silicate (B) corresponds to the peak at 32.6°. Both compounds are essential for the strong development of the mortar matrix [48,49]. The variation in peak intensity is associated with differences in crystallinity and amorphous content among the compounds. The portlandite peak showed a more reduced peak intensity with 0.4% and 0.1% MC compared to CNC, likely due to the enhancement of CNC, which improves interactions with lime and silica in the OPC mix. This facilitates the formation of key hydration products (C3S and C2S), which are crucial for strength development. These findings align with previous studies on Portland cement pastes [26,50].

In addition, the crystallinity index of MC was found to be 9.6% lower than that of CNC due to the higher content of amorphous regions [31], as shown in Table 4. Similarly, the addition of MC and CNC at contents of 0.1% and 0.4% to OPC resulted in increased crystallinity compared to the control OPC, with the CrI reaching 52.2% at P-CNC0.4. The enhanced crystallinity of the CNC-reinforced cement composites, combined with proper dispersion, contributed to their increased strength, which is consistent with the compressive strength results presented in Figure 3.

## 4. Conclusions

This study demonstrates the potential of bamboo-derived cellulose materials, specifically CNC and MC, as effective reinforcement agents in OPC composites. The addition of MC fibers resulted in a decrease in compressive strength as the MC content increased from 0.1% to 0.6%, with higher concentrations causing agglomeration and increased porosity, which weakened the material’s ability to withstand compressive loads. In contrast, the addition of CNC at 0.1% and 0.2% caused slight reductions in compressive strength due to poor dispersion. However, the 0.4% CNC mixture exhibited the highest compressive strength by improving matrix density and reducing porosity. The optimal CNC content for enhancing compressive strength was found to be 0.4%, as it interacted effectively with the cement matrix, improving dispersion and preventing agglomeration. Higher CNC concentrations (0.6%) caused agglomeration, leading to reduced strength due to poor dispersion.

Regarding flexural strength, the addition of MC had a slight positive effect, with a small increase in strength from 7.5 MPa to 8.1 MPa across the range of MC concentrations (0.1% to 0.6%). CNC initially enhanced flexural strength at 0.1%, but this effect declined as the concentration increased. At higher concentrations (0.4% and 0.6%), the flexural strength decreased due to increased brittleness, caused by the high crystallinity of CNC and agglomeration. MC significantly increased the load versus deflection and flexural toughness, particularly at 0.6%, by preventing brittle failure. CNC exhibited better toughness than MC at lower concentrations (0.1%) due to its nanoscale properties, which contributed to better stress transfer and crack arrest.

Microstructural analysis using SEM and FTIR revealed that MC increased the porosity of the cement composites, especially at higher concentrations, while CNC at 0.4% improved the cement matrix by reducing porosity. CNC also demonstrated more homogeneous dispersion and better interaction with the cement matrix, enhancing the mechanical properties. These findings suggest that the optimal use of CNC at 0.4% and MC at lower concentrations can effectively improve the mechanical properties of OPC composites, particularly in terms of compressive and flexural strength, as well as toughness. However, higher concentrations of both materials led to agglomeration, negatively affecting the composite’s performance. These results underscore the potential of bamboo-derived cellulose materials in improving cementitious composite performance, providing a sustainable approach for future construction materials.

## Figures and Tables

**Table 1:** Composition and conditions for sample production.

Reference	Cement Matrix	MC Content (%)	CNC Content (%)	w/c	Curing Temperature (°C)
P-MC0.1	OPC	0.1	-	0.3	25
P-MC0.2	OPC	0.2	-	0.3	25
P-MC0.4	OPC	0.4	-	0.3	25
P-MC0.6	OPC	0.6	-	0.3	25
P-CNC0.1	OPC	-	0.1	0.3	25
P-CNC0.2	OPC	-	0.2	0.3	25
P-CNC0.4	OPC	-	0.4	0.3	25
P-CNC0.6	OPC	-	0.6	0.3	25
OPC	OPC	-	-	0.3	25

**Table 2:** Dimensional analysis of MC and CNC derived from bamboo.

Type of Fibers	Diameter (nm)	Length (nm)
MC	14.6 × 10^3^ ± 4.6	75.2 × 10^3^ ± 31.7
CNC	29.3 ± 10.3	212.2 ± 107.7

Mean ± standard deviation values.

**Table 3:** Flexural toughness parameters and maximum load versus deflection of composite cement.

Samples	Toughness (N mm)	Maximum Load (N)	Deflection (mm)
OPC	302.3	3216.0	0.3
P-MC0.1	406.5	2936.1	0.3
P-MC0.2	454.3	2945.9	0.4
P-MC0.4	711.0	3477.0	0.5
P-MC0.6	992.9	3526.0	0.6
P-CNC0.1	900.4	3504.3	0.8
P-CNC0.2	586.2	3260.4	0.5
P-CNC0.4	247.1	3105.1	0.2
P-CNC0.6	125.0	2528.5	0.2

**Table 4:** Crystallinity index (CrI) of OPC control, MC, and CNC.

Samples	MC	P-MC0.1	P-MC0.4	CNC	P-CNC0.1	P-CNC0.4	OPC
CrI (%)	42.6	47.7	47.6	52.2	49.2	52.1	38.9

## Data Availability

The original contributions presented in this study are included in the article. Further inquiries can be directed to the corresponding authors.
